# Cow's milk entheropathy

**DOI:** 10.1186/1939-4551-8-S1-A271

**Published:** 2015-04-08

**Authors:** Isaac Azevedo Tenorio, Aderbal Sabra, Selma Sabra

**Affiliations:** 1 Unigranrio, Brazil

## Objective

To study the main clinical manifestations and laboratory findings presented by patients with cow's milk enteropathy (CME).

## Material and Methods

Medical records of 40 children and adolescents were studied, 21 males and 19 females with a mean age of 4, 2625 years (maximum: 12 years / Min: 2 months) with the diagnosis of food allergy and CME.

## Results

Chief Complain: 1 - weightloss (16-40%), 2 - abdominal pain (14-35%), 3 - diarrhea (13 - 32.5%), 4 – associated respiratory complains (12-30% ), 5 - vomiting (7 - 17.5%). Other clinical Picture with less ferquency: constipation and abdominal distension.

Malt systemn affected and Homing response: GALT - 40 (100%), BALT - 30 (75%), SALT - 28 (70%), CNSALT - 18 (45%)

Clinical main manifestations in each organ of shock: GALT: Diarrhea (19 to 47.5%), abdominal pain (15 - 37.5%), bulkystools (14-35%), lack of appetite (12-30%). Other less frequent: vomiting, reflux, bloating and abdominal distention. BALT: Rhinitis (11-36.66%), asthma / bronchitis (10 - 33.33%), pharyngotonsillitis (9-30%), phlegm (9-30%). Other less frequent: otitis, sinusitis, snoring and coughing. SALT: Facial pale (18 - 64.29%), shiners (9 - 32.14%), prurigoestrófilo (8 - 28.57%), atopic eczema (6-21,42%). Other less frequent: urticaria and erythemaperianal. CNSALT: Irritability (9- 50%), sleepdisorder (7- 38.88%), Hyperactivity (4- 22.22%), headache (3- 16.66%). Other less frequent: ADHD and fatigue.

## Laboratory tests

IgE: IgE increased: 20 (52.63%), the normal IgE: 18 (47.36%). (2 patients IgE Unknown); Ratio CD4 / CD8: CD4 / CD8 ratio was low in 16 cases (43.24%), CD4 / CD8 ratio was high in 10 cases (27.07%) and CD4 / CD8 ratio was Normal: 11 (29.72% ). (3 patients with CD4 / CD8 ratio unknown) .IGG4 greater than IgG3: normal relationship: IgG3>IgG4 in 20 cases (60.6%); ratio reversed: IgG4>IgG3 in 13 cases 13 (39.4%) and 7 patientswith IgG3 and IgG4 Unknown.

## Conclusion

This study adds important clinical data to the understanding of cow's milk enteropathy. In our experience this clinical entity, occurs at any age, from infancy to adolescence and affect both sexes.

